# The impact of self-stigmatization on the mental health of female sex workers (FSWs)

**DOI:** 10.3389/fpubh.2025.1679876

**Published:** 2025-11-13

**Authors:** Gizem Kaya, Olivia Kalinowski, Franziska Kroehn-Liedtke, Anastasiia Lotysh, Hristiana Mihaylova, Lena Zerbe, Wulf Rössler, Meryam Schouler-Ocak

**Affiliations:** Department of Psychiatry and Neurosciences, Psychiatric University Clinic of Charité at St. Hedwig Hospital, Berlin, Germany

**Keywords:** female sex workers, self-stigmatization, mental health, stigma mechanisms, affective disorders, trauma-related disorders

## Abstract

**Introduction:**

Sex workers are exposed to high levels of mental health risk. Yet, the psychological effects of self-stigmatization in legalized sex work contexts remain underexplored. This study examines how different dimensions of self-stigma influence mental health outcomes among female sex workers (FSWs) in Germany, where sex work is legalized and regulated.

**Methods:**

A cross-sectional study was conducted with 397 FSWs recruited across diverse work settings in Germany between August 2022 and October 2024. Mental health conditions were assessed using the Mini-DIPS Open Access structured interview. Self-stigmatization was measured via the Paradox of Self-Stigmatization Scale (PaSS-24), which captures three dimensions: *stereotype endorsement*, *non-disclosure*, and *righteous anger*. Logistic regression analyses were used to examine associations between self-stigma and four psychiatric outcomes: affective disorders, anxiety disorders, trauma-related disorders, and substance use disorders, adjusting for demographic and occupational covariates.

**Results:**

A high prevalence of mental health disorders was observed among participants, reflecting the categories assessed in this study (affective, anxiety, trauma-related, and substance use disorders). Emotional and behavioral dimensions of self-stigmatization, particularly concealment and emotional reactivity, showed associations with certain psychiatric outcomes. In contrast, cognitive endorsement of stereotypes showed no consistent links to mental health status in this sample.

**Discussion:**

Findings support the “Paradox of Self-Stigma” model: FSWs cognitively reject negative stereotypes yet exhibit strong emotional and behavioral responses that heighten psychological distress. *Righteous anger* and concealment may reflect unresolved trauma rather than resilience. Interventions should address emotional stigma responses, promote safer work environments, and support disclosure in trusted relationships. This study highlights the need for context-sensitive, multidimensional strategies to reduce stigma-related mental health burdens among sex workers in legalized systems.

## 1 Introduction

Sex work refers to the exchange of sexual services for payment and has existed throughout history in various forms, including street-based sex work, brothel work, escort services, dominatrix services, and pornographic modeling (1). It encompasses both direct physical contact, such as sexual intercourse, and indirect sexual stimulation, such as bondage or lap dancing (2). When examining sex work, it is crucial to consider that it is closely linked with criminal concerns such as human trafficking, coerced prostitution, and organized crime (3). Differentiating between consensual and forced sex work is often challenging, which complicates legal processes and provision of access to health care (3). The legal status of sex work varies worldwide, reflecting differing societal attitudes on issues such as gender inequality and sexual exploitation (4, 5). In Germany, sex work has been classified as a legal form of labor since 2002 and is regulated under the Prostitute Protection Act of 2017, which aims to improve the legal and social conditions of sex workers (6). Under this law, sex workers must register their activity and obtain a registration certificate. According to the German Federal Office of Statistics, 30,600 sex workers were officially registered in Germany in 2024, with only 18% being German citizens (7). Estimates of the number of sex workers in Germany vary widely. Research-based estimates range between 64,000 and 200,000 sex workers (7–9). Scholars argue that, despite legal requirements, fear of stigmatization and social exclusion may discourage many from registering, explaining why only a small fraction of sex workers appear in the official statistics (10, 11).

Stigma is a socio-cultural process that labels certain groups as undesirable (12). It exists in two forms: enacted stigma, also referred to as external stigma, and self-stigma, also known as internal stigma. Enacted stigma refers to discrimination and unfair treatment by others, while self-stigma occurs when individuals internalize negative societal stereotypes, leading to feelings of shame, self-discrimination, and expectation of rejection (13, 14). According to the stage model of self-stigma, individuals first become aware of public stigma, then internalize these negative stereotypes, and ultimately apply them to themselves (14). Self-stigma represents an internalization of public stereotypes that erodes self-esteem and self-efficacy, ultimately contributing to depression, anxiety, and other mental health problems. Individuals who internalize stigma often withdraw socially and avoid discussing their experiences or seeking professional help, which further reinforces psychological distress and limits access to healthcare services (15, 16). A large body of research has demonstrated the detrimental impact of self-stigma on well-being, including reduced hope, empowerment, and quality of life (5, 17–21).

Stigma-research has traditionally focused on minority migrants, physical health conditions, drug use, disability, chronic diseases such as diabetes, and individuals with mental health disorders such as schizophrenia (17, 22–24). The concept of intersectional stigma has emerged, describing multiple stigmas experienced by a person or group, which are associated with worse health behaviors and outcomes, including more severe symptoms of depression (25). Self-stigma results in an overall decline of quality of life (i.e., subjective well-being) and a self-esteem (16, 21), both of which negatively influence mental health by increasing vulnerability to depression and anxiety (19, 21). Furthermore, self-stigma leads to social withdrawal and prevents people from discussing their experiences, seeking support, or utilizing healthcare services (5, 13). Research has shown that individuals who internalize stigma and anticipate discrimination from healthcare providers, are less likely to access medical care or adhere to follow-up appointments (12, 15, 26).

Female sex workers (FSW) constitute the largest subgroup within the sex worker industry (27). They experience significantly higher rates of mental health disorders, including depression, anxiety, post-traumatic stress disorder (PTSD) and substance abuse, compared to the general population (27–31). Empirical evidence from large-scale studies in East Asia has shown that self-perceived stigma and experiences of violence are consistently linked to depressive symptoms, anxiety, and suicidal behaviors among FSWs, underscoring stigma as a central psychological mechanism in this population (32, 33). Contributing factors include economic and social vulnerabilities such as age, lack of social support, and migrant status and occupational stressors such as unsafe working conditions, exposure to violence, and discrimination (27, 34, 35). Additionally, FSWs often belong to multiple marginalized groups (e.g., migrants, racialized minorities), which exposes them to intersectional stigma, further worsening their mental health outcomes (5, 23, 36, 37). Existing studies often focus on sex workers affected by human immunodeficiency virus (HIV)- and sexually transmitted infection (STI)-related stigma and its effect on healthcare access (38–46). A systematic review found stigma to be a moderating variable that increased the severity of mental health symptoms among sex workers (27). Despite growing recognition of the role of stigma for mental health, research gaps remain. There is currently no quantitative study examining the impact of self-stigma on the mental health of sex workers in Germany. Given the country’s legal framework and the high proportion of migrant female sex workers, it is crucial to understand how self-stigma affects their mental well-being in different working environments.

This study addresses this gap by conducting a cross-sectional quantitative analysis on the relationship between self-stigma and mental health outcomes among female sex workers. The findings aim to inform targeted interventions that reduce stigma and improve healthcare accessibility for this marginalized population. We hypothesized that emotional (*righteous anger*) and behavioral (*non-disclosure*) dimensions of self-stigma would be positively associated with affective and trauma-related disorders, whereas cognitive endorsement (*stereotype endorsement*) would show weaker associations. To address these aims, the study investigated the following research questions:

- Does *stereotype endorsement* influence the prevalence of mental disorders among FSWs?
- Does *righteous anger* influence the prevalence of mental disorders among FSWs?
- Does *non-disclosure* influence the prevalence of mental disorders among FSWs?

## 2 Methods

### 2.1 Participants

Participants were eligible if they identified as female and had engaged in sex work (providing sexual services in exchange for money or goods) in any setting (brothels, hotels, apartments, studios, as escorts, street-based or online). Only participants over the age of 18 were included. Participants were eligible if they spoke German, English, Vietnamese, Turkish, Hungarian, Romanian, Polish, Russian, Ukrainian, or Bulgarian. Individuals who were unwilling or unable to provide informed consent due to cognitive impairment or language barriers were excluded from the study.

### 2.2 Instruments and materials

To assess self-stigmatization, the Paradox of Self-Stigmatization Scale (PaSS-24) by Golay et al. (14) was used. We selected the PaSS-24 because it captures three distinct yet interrelated domains of self-stigma:

- *Stereotype endorsement* (agreement) – the extent to which individuals accept negative societal stereotypes about their own group.
- *Non-disclosure* (concealment) – the tendency to hide one’s affiliation with a stigmatized group.
- *Righteous anger* (paradoxical empowerment) – the expression of anger as a response to stigmatization.

Thus, allowing for multidimensional understanding of stigma-related mechanisms. Compared to other instruments, the PaSS-24 offers strong psychometric properties and cultural flexibility. A validation study by Golay et al. (14) explicitly compared it with alternative scales and confirmed its utility in differentiating between cognitive, emotional, and behavioral stigma reactions. The original version of the PaSS-24 was translated into German and further adapted into several additional languages commonly spoken among the participants following a standardized forward-backward translation procedure. The translated versions were reviewed by bilingual psychologists at the Department of Psychiatry, to ensure semantic and conceptual equivalence. The PaSS-24 has demonstrated good internal consistency in the validation study by Golay et al. (14), with Cronbach’s *α* = 0.84 for *stereotype* endorsement, 0.83 for *non-disclosure*, and 0.81 for *righteous anger* (*α* = 0.89 for the total scale). Convergent validity was supported by strong correlations with established stigma measures (*r* = 0.71, *p* < 0.001), and confirmatory factor analysis confirmed its three-factor structure (CFI = 0.94, RMSEA = 0.05). Each of the three dimensions of the PaSS-24 (*stereotype endorsement, non-disclosure, and righteous anger*) comprises eight items rated on a 5-point Likert scale (1 = strongly disagree, 5 = strongly agree), yielding possible subscale scores ranging from 8 to 40. Understanding the numerical values is critical for interpreting the psychological meaning of self-stigma in this population. To facilitate interpretation, subscale scores were categorized into three levels: low (8–18), moderate (19–29), and high (30–40), with higher scores indicating stronger expression of the self-stigma dimensions *righteous anger* and *non-disclosure*. For example, a score of 30 on *righteous anger* reflects a high emotional response to stigma, suggesting that the participant strongly reacts to injustice and marginalization. Conversely, a score of 14 on *stereotype endorsement* implies a low degree of internalized negative belief, meaning participants largely reject prevailing stereotypes about sex workers. While low endorsement of stereotypes may offer some cognitive protection, high scores on *non-disclosure* and *righteous anger* have been shown to correlate with mental health burdens. This underscores the need to treat self-stigma as a multidimensional construct: cognitive rejection of stigma may coexist with emotional distress and avoidance behavior, which are more directly linked to adverse mental health outcomes. In line with the study’s objectives, analyses were conducted separately for each subscale; no total score was calculated, as the three dimensions capture theoretically distinct components of self-stigma. This approach allows for a more differentiated understanding of how specific aspects of self-stigma relate to mental health outcomes.

Mental disorders were assessed using the Mini-DIPS Open Access by Margraf and Cwik (47), a structured diagnostic interview that allows trained non-clinicians to reliably assess lifetime and current mental disorders according to DSM-5 and ICD-10. The Mini-DIPS Open Access was chosen because it provides a standardized diagnostic evaluation, has demonstrated high inter-rater reliability, and can be applied by trained non-clinicians. Alternative screenings tools were considered but rated less suitable, as they do not provide clinically robust diagnostic categories and would have limited the depth of this study. The Mini-DIPS Open Access shows excellent psychometric properties, with inter-rater reliability between *κ* = 0.88 and 0.98 and test–retest reliability between *κ* = 0.80 and 0.91 (47). Criterion validity was confirmed through high agreement with the SCID-5 (*κ* ≈ 0.84) and sensitivity/specificity indices above 0.85 (47). For the purpose of analysis, only affective disorders, anxiety disorders, trauma-related disorders, and substance use disorders were considered, as these were central to the research question. Other diagnostic categories assessed by the instrument were excluded from further analysis. These diagnostic categories were selected because they represent the most prevalent and clinically relevant mental health conditions among female sex workers, as consistently reported in previous research (28). Moreover, these disorders are closely linked to processes of stigmatization and self-stigmatization, making them particularly pertinent for examining the relationship between self-stigma and mental health. Other diagnostic categories assessed by the Mini-DIPS (e.g., eating or somatoform disorders) were excluded due to their low prevalence in this sample and limited theoretical relevance to the study’s objectives (28).

In addition to the PaSS-24 for self-stigmatization and the Mini-DIPS Open Access for mental disorder assessment, we collected data on sociodemographic factors and work conditions through self-reported responses based on the Sex-Work-Questionnaire by Rössler et al. (2010) and adapted it linguistically to the target population. The questionnaire included items covering age, education, migration background, income, type of sex work, and disclosure status. Items were mostly categorical, with work-type responses allowing multiple selections. The questionnaire was specifically designed for this study to capture contextually relevant aspects of sex work in Germany. It was developed in consultation with researchers and practitioners experienced in working with sex workers but was not formally validated. Therefore, psychometric indices such as reliability and validity are not available. Only items relevant to the present analyses were included. A shortened English version of the sociodemographic and occupational items used in this study is provided in the Supplementary Appendix B1. Items were answered in the participant’s preferred language (German, English, Turkish, Romanian, Polish, Vietnamese, Russian, Ukrainian, Hungarian, or Bulgarian).

### 2.3 Procedure

Selecting a random, probabilistic sample was infeasible due to the mostly invisible and mobile nature of the target group. Therefore, a non-proportional quota-sampling was used to ensure representation of various work settings and language proficiencies. Both independent and venue-affiliated workers were recruited. Particular attention was paid to including different “types” of sex work, such as street-based, brothel-based, escort, dominatrix, online, hotel-based, and apartment-based work. This approach ensured that the sample reflects the heterogeneity of sex work in Germany and allows for conclusions that extend beyond one occupational subgroup. Participants worked across multiple settings, reflecting the heterogeneity of sex work in Germany. Of the total sample (*N* = 397), 29.97% worked in clients’ apartments, 30.48% online, 52.14% in hotels, 22.17% in brothels, 37.28% in their own apartments, 29.22% in street-based contexts, and 25.44% via escort services. Percentages exceed 100% because participants could report multiple concurrent settings. Although these data appear in the Results (Table 1), we summarize them here to provide a complete description of the sample. The relative contribution of each recruitment channel was not systematically recorded. However, efforts were made to ensure a diverse and heterogeneous sample by targeting different outreach setting and sex work contexts. Participants were initially recruited through walk-in and advice centers in Berlin by distributing multilingual flyers to their clients, which informed potential participants about procedures and aims of the study. In further steps, members of various autonomous groups of sex work activists were contacted. Study employees regularly attended events organized by these groups and conducted recruitment interviews. In addition, participants were recruited at Germany’s largest annual erotic fair in 2022 and 2023. Participants were also recruited in brothels and dominatrix studios. The operators of various prostitution sites were contacted with a request for permission to conduct the study on their premises (the interviews took place in separate rooms between the participant and the study employee). Potential participants were also contacted online by study staff on various escort and community sites (such as OnlyFans). Recruitment was also carried out by contacting practicing gynecologists and psychiatric institutions with the request that they pass on the study information to eligible women. Therefore, every major occupational subtype of female sex work was represented in the final sample, providing a heterogeneous and contextually comprehensive picture of this population. However, despite these extensive recruitment efforts, it cannot but fully ruled out that certain highly hidden subgroups remain underrepresented. Because participation relied partly on outreach through service organizations and public events, women working in extreme isolation or without access to such networks may have been less likely to participate. The overall response rate could not be calculated because the number of individuals approached through different recruitment channels was not systematically recorded. This limitation reflects the open and community-based recruitment strategy used in this study, which prioritized accessibility and anonymity for participants over formal tracking of contacts.

**Table 1:** Descriptive statistics of the sample.

Variable	Value	*n*
Age (mean ± SD)	33.85 ± 9.19	397
Age (median)	32	
Age range	19–70	
Migration background	61.21%	243
German citizenship	58.94%	234
German language skills	82.62%	328
School education	85.39%	339
Secondary vocational education	60.96%	242
Currently engaged in sex work	85.89%	341
Was forced into sex work	10.58%	42
Sex work main source of income	68.01%	270
The partner is aware of the sex work	39.04%	155
Social contacts are aware of the sex work	61.21%	243
Sex work without the use of an alias	15.11%	60
Work setting: client’s apartment	29.97%	119
Work setting: online	30.48%	121
Work setting: hotel	52.14%	207
Work setting: brothel	22.17%	88
Work setting: own apartment	37.28%	148
Work setting: street-based	29.22%	116
Work setting: escort	25.44%	11

The data collection for the present study was carried out between August 2022 and October 2024 in collaboration with the Psychiatry Department at Charité University Hospital in Berlin and as part of the study titled “Mental health in sex workers: A cross-sectional survey” funded by the German Research Association. The study design and methodology were examined and confirmed by the Charité Ethics Committee in (21.09.2020)/ (EA2/133/18), implementing measures to ensure participants’ confidentiality and privacy. Participants were informed about their right to withdraw their data at any point. Eligible and consenting recruits participated in an anonymous face-to-face survey conducted in person or via video call with trained staff, fluent in various languages (German, English, Vietnamese, Turkish, Bulgarian, Polish, Russian, Ukrainian, Hungarian, and Romanian). All participants received a 50 Euro compensation upon completion. The face-to-face interview took 60 to 90 min. No participants reported acute distress during interviews. Trained staff were prepared to pause or terminate interviews and provide referral to mental health services if participants exhibited discomfort. In addition, a dedicted consultation service was established at the Department of Psychiatry, offering information and guidance on how to access psychotherapeutic support. Participants were informed that they could contact this service after completing the interview. Only one participant made use of this opportunity and attended a single counseling session.

### 2.4 Data analysis

Statistical analyses were conducted using IBM SPSS 29. The dependent variable was the point prevalence of mental disorders. Logistic regressions were performed to examine the association between self-stigmatization dimensions – *stereotype endorsement, righteous anger, non-disclosure* and the outcome variables anxiety disorders, affective disorders, trauma-related disorders, and substance use disorders. All predictors in the logistic regression models were either dichotomous or quasi-metric. Therefore, no pairwise comparisons were required, as each coefficient represents the contrast inherent to the predictor. A hierarchical approach was used to identify the relevant influencing factors during model development. In the first step, the data were adjusted for relevant sociodemographic confounders including age, educational level and migration background. From the remaining influencing factors, statistically significant variables were identified in a subsequent analysis step using a stepwise variable selection procedure. Model quality was evaluated using Nagelkerke’s *R*^2^, the Hosmer-Lemeshow test, and ROC curve analysis. Odds ratios (ORs) with 95% confidence intervals (CIs) were reported. Statistical significance was defined at *p* ≤ 0.05.

## 3 Results

The following sections presents the descriptive and analytical findings of the study. The results are presented in three main tables summarizing the key descriptive and analytical findings.

### 3.1 Participants characteristics

Table 1 provides an overview of the sociodemographic and occupational characteristics of the sample, illustrating the diversity of working contexts represented in this study. Detailed subgroup distributions of self-stigma dimensions by demographic characteristics are available in the Supplementary Tables S1,S2. A total of 397 participants (98.5% of the full sample, *N* = 403) were included in the final analysis, having provided complete and valid data on all relevant variables. The mean age of the sample was 33.85 years (*SD* = 9.19; range: 19–70). A substantial proportion (61.21%) reported a migration background (Table 1). The data indicate that FSWs often operate across multiple settings rather than being limited to just one environment. Participants reported working in clients’ homes (30%), online (30.5%), in hotels (52.1%), in brothels (22.2%), their own apartment (37.3%), on the street (29.2%), and through escort services (25.4%). These overlapping percentages highlight the fluid nature of their work.

In addition to the regression results, descriptive analyses of the PaSS-24 subscales revealed distinct response patterns within the sample (*N* = 397) (Supplementary Table S1). Participants showed substantial variation across the three self-stigma dimensions, with particularly high values observed for *righteous anger* and lower values for *stereotype endorsement*. A total of 62.2% of participants reported high levels of *righteous anger*, indicating strong emotional responses to perceived stigma and social injustice. Similarly, 29.7% scored high on *non-disclosure*, suggesting that nearly one-third of the sample consistently concealed their occupational identity. In contrast, only 2.3% showed high *stereotype endorsement*, while 77.8% scored in the low range, indicating a clear cognitive rejection of stigmatizing beliefs about sex work. This descriptive pattern supports the theoretical framework of the “Paradox of Self-Stigmatization,” in which individuals cognitively reject negative stereotypes while still experiencing strong emotional and behavioral responses that reflect anticipated discrimination.

In addition to overall prevalence, self-stigma dimensions differed systematically across sociodemographic groups (Supplementary Table S2):

- *Righteous anger*: Younger FSWs (<30) were the most likely to express high levels of anger in response to stigma (72.8%), compared to 55.3% (ages 31–40) and 51.8% (ages 41+). Similarly, high *righteous anger* was more prevalent among those with migrant background, lower education, and lower income. For example, 75.8% of those without secondary schooling scored high on *righteous anger* versus 58.2% of those with schooling (*χ*^2^, *p* < 0.001).
- *Non-disclosure*: Concealment was highest among middle-income FSWs and those with moderate education, but less clearly stratified by age or citizenship. Roughly 29.7% of the total sample scored high on *non-disclosure*, with migrants and lower-earning participants overrepresented in this category. Notably, women earning above €3,000 reported much lower *non-disclosure* scores, suggesting greater comfort with disclosure under conditions of economic security.
- *Stereotype endorsement*: As reported previously, *stereotype endorsement* was significantly lower among participants with higher income, education, and German citizenship. These cognitive aspects of stigma were most stratified by structural positioning.

Together, these findings indicate that emotional and behavioral responses to stigma (*righteous anger* and *non-disclosure*) are not randomly distributed but follow clear social gradients, much like cognitive internalization (*stereotype endorsement*).

### 3.2 Prevalence of mental disorders

Table 2 presents the prevalence of current mental disorders, highlighting the high rates of affective, anxiety, trauma-related, and substance use disorders among female sex workers. A total of 74,6% of participants were affected by at least one mental disorder at the time, based on the conditions assessed, with the most frequently diagnosed conditions being: affective disorders (55.2%), anxiety disorders (45.6%), followed by trauma-related disorders (34.3%) and substance use disorders (27.7%) (Table 2).

**Table 2:** Prevalence of mental disorders.

Mental disorder	Prevalence (%)
Anxiety disorder	45.6%
Affective disorder	55.2%
Trauma-related disorder	34.3%
Substance use disorder	27.7%

### 3.3 Associations between self-stigma and mental health

Logistic regression analyses were conducted to assess the relationship between dimensions of self-stigmatization and mental health outcomes, while controlling for sociodemographic and occupational variables using a hierarchical model approach. Table 3 summarizes the results of the logistic regression analyses examining associations between self-stigma dimensions and mental health outcomes, adjusted for sociodemographic and occupational variables. Additional model diagnostics and contextual predictors are reported in the Supplementary Tables S3,S4 to improve clarity, while the main text focuses on the findings most relevant to the research aim. Statistical significance was defined at *p* ≤ 0.05 (Table 3).

- Affective disorders were significantly associated with *righteous anger* [OR = 1.05, 95% CI (1.01, 1.08), *p* = 0.005], suggesting internalized emotional responses to stigma play a role in affective pathology.
- Trauma-related disorders were significantly predicted by both *righteous anger* [OR = 1.06, 95% CI (1.02, 1.10), *p* = 0.004] and *non-disclosure* [OR = 1.06, 95% CI (1.01, 1.10), *p* = 0.013], indicating that concealment and affective reactions to stigma contribute to the likelihood of trauma-related symptomatology.
- Anxiety and substance use disorders did not show statistically significant associations with the measured dimensions of self-stigma within this sample. For anxiety disorders, the OR for *righteous anger* was 1.02 [95% CI (0.99, 1.06), *p* = 0.261], for *non-disclosure* 1.01 [95% CI (0.98, 1.05), *p* = 0.621], and for *stereotype endorsement* 1.02 [95% CI (0.99, 1.05), *p* = 0.082]. For substance use disorders, *righteous anger* had an OR of 1.02 [95% CI (0.98, 1.06), *p* = 0.325], *non-disclosure* OR = 1.03 [95% CI (0.99, 1.07), *p* = 0.187], and *stereotype endorsement* OR = 1.01 [95% CI (0.97, 1.05), *p* = 0.550].

**Table 3:** Logistic regression results (selected predictors).

Outcome	Predictor	OR 95% CI	*p*-value
Affective disorder	Righteous anger	1.05 (1.01, 1.08)	0.005
Trauma-related disorder	Righteous anger	1.06 (1.02, 1.10)	0.004
Trauma-related disorder	Non-disclosure	1.06 (1.03, 1.09)	<0.001
Anxiety disorder	Non-disclosure	1.01 (0.98, 1.05)	0.621
Anxiety disorder	Righteous anger	1.02 (0.99, 1.06)	0.261
Anxiety disorder	Stereotype endorsement	1.02 (0.99, 1.05)	0.082
Affective disorder	Non-disclosure	1.02 (0.99, 1.06)	0.141
Affective disorder	Stereotype endorsement	1.00 (0.97, 1.03)	0.988
Trauma-related disorder	Stereotype endorsement	1.01 (0.96, 1.06)	0.661
Substance use disorder	Non-disclosure	1.03 (0.99, 1.07)	0.187
Substance use disorder	Righteous anger	1.02 (0.98, 1.06)	0.325
Substance use disorder	Stereotype endorsement	1.01 (0.97, 1.05)	0.550

Model diagnostics indicated good model fit across all outcomes, with Nagelkerke’s *R^2^* values ranging from 0.17 to 0.22 and area under the curve (AUC) values exceeding 0.70, suggesting acceptable to good discriminative ability (Supplementary Table S3).

### 3.4 Environmental and demographic risk factors

Several contextual and demographic variables emerged as significant predictors across the models after adjusting the data for relevant sociodemographic and environmental cofounders within this sample (Supplementary Table S4). Working in the client’s apartment was associated with increased odds for all four mental health outcomes. Disclosure of one’s sex work to a partner was associated with a reduced risk of mental disorders, whereas disclosure to social contacts outside of sex work was associated with increased risk. Age showed a protective effect across models, while German nationality and higher educational attainment were linked to higher odds of anxiety and affective disorders. These variables were not significant predictors in the substance use disorder model in this sample.

## 4 Discussion

This study aimed to explore the impact of self-stigmatization on the mental health of female sex workers (FSWs) in Germany. The descriptive findings on the PaSS-24 subscales offer further insight into the complexity of self-stigmatization processes in this population. Sociodemographic stratification in self-stigma dimensions confirms the complex interplay between structural vulnerability and psychological coping. While cognitive stigma rejection (low *stereotype endorsement*) is strongly linked to social privilege (German nationality, education, income), emotional (*righteous anger*), and behavioral (*non-disclosure*) components appear more reactive to lived marginalization. Younger sex workers and those from migrant backgrounds were more likely to experience *righteous anger*—a pattern that may reflect chronic exposure to exclusion, injustice, or internal ambivalence. Similarly, concealment (*non-disclosure*) remained prevalent across subgroups but was particularly elevated in women with unstable income and precarious legal status. This concealment may serve as an adaptive strategy to avoid social sanctions, yet comes at a psychological cost, especially in contexts where stigma is anticipated but not cognitively internalized. These findings reinforce the theoretical model of “paradoxical self-stigma,” in which individuals cognitively reject stereotypes while still experiencing emotional and behavioral strain. Interventions must acknowledge that stigma responses differ not only by individual disposition but also by socioeconomic position. Stigma-reduction programs must therefore include economic empowerment, legal security, and culturally grounded peer-support mechanisms as core components, especially for marginalized subgroups within the sex worker population. However, they simultaneously reported high levels of *righteous anger* and *non-disclosure.* Individuals may reject public stigma on a cognitive level but still act cautiously (concealment) or defensively (anger) in anticipation of judgment or exclusion. These results suggest that interventions focusing solely on changing internalized beliefs may not be sufficient; instead, strategies must address emotional regulation and create safer conditions for disclosure.

Our findings align with previous research documenting the mental health burden among sex workers. A recent systematic review identified consistently high rates of depression, anxiety, and suicidality across global samples of female sex workers, supporting the high prevalence observed in our study (27). Likewise, research from diverse contexts, including Kenya (37) and China (32, 33) has shown, that stigma, exposure to violence, and unsafe working conditions are strongly associated with depressive and trauma-related symptomatology. The convergence of our results with these findings suggests that the paradoxical pattern we observed (cognitive rejection of stereotypes yet high emotional and behavioral reactivity) may represent a cross-contextual mechanism linking stigma to poor mental health outcomes, even in legalized systems such as Germany’s. By embedding our results within this established evidence base, the present study extends prior literature by specifying the psychological pathways through which stigma translates into distress. It is not only the presence of stigmatizing beliefs that predicts mental health problems, but rather their emotional and behavioral manifestation (concealment and anger) that appear to mediate the association with trauma-related and affective disorders. These insights highlight the need for interventions that address the emotional and behavioral consequences of stigma, in addition to challenging public stereotypes themselves.

Occupational setting emerged as the most robust environmental risk factor in this sample. Working in a client’s apartment increased risk across all psychiatric domains, likely due to reduced autonomy, poor boundary control, and heightened exposure to violence, findings that echo previous reports of street-based or unregulated sex work correlating with poorer mental health. Conversely, escort work, which often offers more autonomy and selective client interactions, was associated with reduced risk. Disclosure dynamics added further complexity. While openness with intimate partners buffered psychological distress, consistent with the stress-buffering hypothesis, being known as a sex worker in broader social circles correlated with elevated psychiatric risk, likely due to external discrimination and social exclusion. Interestingly, German nationality and higher education were associated with higher risk for anxiety and mood disorders. This may reflect internalized dissonance, where women from more privileged or culturally integrated backgrounds experience greater identity conflict due to normative violations inherent in sex work. *Non-disclosure* was also found to be significantly linked to poor mental health outcomes. FSWs who feel the need to lead a double life may experience anxiety about exposure, strained relationships, or a lack of social support, all of which contribute to mental health problems. However, further research is needed to explore whether *righteous anger* leads to actual empowerment or if it masks deeper emotional distress. This suggests that the mere cognitive rejection of societal stereotypes may be less influential on psychological distress than emotional or behavioral reactions to stigma. Understanding which components (cognitive, emotional, or behavioral) are most relevant for specific outcomes can guide the development of more targeted and effective interventions. These findings underscore the importance of contextualizing self-stigma within occupational, relational, and structural environments. Interventions must be multidimensional: targeting internalized stigma (e.g., through peer-based empowerment programs), improving work safety, and reducing public discrimination. Healthcare professionals should receive stigma-sensitivity training to reduce anticipated discrimination, including racially motivated discrimination, which migrant FSWs may face in healthcare settings. Given the disproportionate burden on migrant and precariously employed FSWs, mental health policies must account for intersectionality and structural barriers.

While the PaSS-24 was validated in a clinical population (*N* = 202 psychiatric inpatients), the authors did not report descriptive statistics such as subscale means or standard deviations for their samples. As a result, direct quantitative comparisons between our FSW sample and clinical or non-clinical populations are not possible. However, based on the scale structure and the observed high scores in our sample, particularly on *righteous anger*, it appears that emotional and behavioral responses to stigma may be particularly pronounced among FSWs. In the absence of standardized normative data for the general population, our findings can be interpreted through a theoretical lens. Despite operating within a legalized framework in Germany, many sex workers remain socially marginalized. The observed profile, marked by low *stereotype endorsement* but high levels of *non-disclosure* and *righteous anger*, suggests that legal recognition does not necessarily translate into social acceptance. In contrast to other marginalized groups, such as individuals with mental illness or chronic illness, who often internalize stigma cognitively. This may reflect a specific form of anticipated discrimination, where individuals expect to be judged or excluded despite rejecting the underlying stereotypes. Such a discrepancy may be particularly pronounced in occupations like sex work, which are legal yet remain heavily stigmatized in the public discourse.

### 4.1 Strengths and limitations

This study represents one of the largest quantitative assessments of female sex workers (FSWs) in Germany focusing on self-stigmatization and mental health. A key strength lies in its multidimensional approach, incorporating factors such as work setting, migration background, legal status, education, and income, allowing for a nuanced understanding of how self-stigma operates in diverse occupational and social contexts.

Nevertheless, several limitations should be acknowledged. The use of a non-proportional quota sampling strategy ensured representation across work settings but limits generalizability, particularly to FSWs in highly precarious or hidden circumstances who may not access services or networks. A major limitation of this study lies in its cross-sectional design. Because all instruments were administered at the same time point, causality cannot be inferred. It is therefore not possible to conclude whether stigma dimensions lead to mental disorders or whether mental illness increases vulnerability to stigma (a problem of potential reverse causality). Longitudinal research will be essential to clarify the directionality of these associations. Additionally, while we controlled for important confounders such as age, migration background, income, and education, other unmeasured factors (e.g., childhood trauma, social network quality, or current physical health) may have influenced the associations observed. We consider it important to be transparent about this limitation so that readers interpret the results with appropriate caution.

Furthermore, due to the lack of normative data for the PaSS-24, comparisons with general or clinical populations remain limited. This restricts the extent to which the observed stigma levels can be evaluated against broader population patterns.

Self-reported data may be affected by recall and social desirability bias, although the use of a standardized clinical interview (MINI-DIPS) partially mitigates this. Some mental health conditions may have been under detected due to the structured format or participant avoidance. While the study controlled for key demographic and occupational variables, unmeasured factors may have influenced results. Although interviews were conducted in multiple languages, subtle cultural and linguistic variations may have shaped how participants understood and responded to items on self-stigma and emotional experiences. Additionally, Germany’s unique legal context limits the transferability of findings to countries with different regulatory or social frameworks surrounding sex work.

Finally, while ethical safeguards were in place, discussing mental health and stigma may have caused emotional distress in some participants. Future research should incorporate more inclusive recruitment strategies and culturally adapted tools and explore these dynamics using longitudinal and clinically validated approaches.

## 5 Conclusion

This study provides new insights into the relationship between self-stigmatization and mental health among female sex workers. The findings demonstrate that emotional (*righteous anger*) and behavioral (*non-disclosure*) dimensions of self-stigma are strongly associated with affective and trauma-related disorders, while cognitive endorsement (*stereotype endorsement*) shows weaker associations. These results support the multidimensional conceptualization of self-stigma and highlight the paradoxical coexistence of resistance and psychological burden described in the paradox of self-stigmatization model.

Beyond their theoretical contribution, the findings underscore the importance of addressing self-stigma in prevention and treatment programs for sex workers. Interventions should foster open communication, reduce internalized stigma, and strengthen emotional coping strategies to mitigate the psychological consequences of discrimination. Future research should employ longitudinal and cross-cultural designs to further clarify causal pathways and examine protective factors that may buffer the impact of self-stigma on mental health.

## Data Availability

The raw data supporting the conclusions of this article will be made available by the authors, without undue reservation.
