# Supplementary material for: The impact of self-stigmatization on the mental health of female sex workers (FSWs)
Source: Front Public Health. 2025 Nov 13;13:1679876. doi: 10.3389/fpubh.2025.1679876 (PMC12657165; doi:10.3389/fpubh.2025.1679876)
Supplement: Supplementary file 1 [file Data_Sheet_1.docx]

**12 Appendix: Supplementary Tables**

The following supplementary tables provide extended analyses that complement the main results presented in the manuscript. These include detailed descriptive statistics of the self-stigma dimensions, subgroup distributions, model fit indices, and additional contextual predictors.

**12.1 Appendix A**

Table A1. Descriptive Analyses of the PaSS-24 Subscales in the Sample

| Sub-Scale | Total Sample (*N* = 397) | No Diagnosis | Diagnosis |
| --- | --- | --- | --- |
| Stereotype Endorsement (Mean ± *SD*) | 14.11 ± 6.31 | 14.19 ± 6.46 | 14.01 ± 6.15 |
| Righteous Anger (Mean ± *SD*) | 30.51 ± 7.10 | 29.54 ± 7.35 | 31.68 ± 6.62 |
| Non-Disclosure (Mean ± *SD*) | 24.43 ± 7.99 | 23.86 ± 7.96 | 25.53 ± 7.93 |

# Table A2. Distribution of Self-Stigma Dimensions (PaSS-24) by Sociodemographic Subgroups (N=397)

| Sociodemographic Variable | Stereotype Endorsement – Low (%) | Righteous Anger – High (%) | Non-Disclosure – High (%) |
| --- | --- | --- | --- |
| Age < 30 | 85.5 | 72.8 | 32.4 |
| Age 31–40 | 73.0 | 55.3 | 27.7 |
| Age 41+ | 69.9 | 51.8 | 27.7 |
| German Citizenship | 90.2 | 58.3 | 24.2 |
| Non-German Citizenship | 60.1 | 68.1 | 33.8 |
| No Migration Background | 90.3 | 57.6 | 25.3 |
| Migration Background | 70.0 | 66.0 | 32.1 |
| With School Education | 83.2 | 58.2 | 28.1 |
| Without School Education | 46.6 | 75.8 | 32.8 |
| With Vocational Training | 81.8 | 61.2 | 26.4 |
| Without Vocational Training | 71.6 | 63.7 | 31.4 |
| Income < €500 | 45.8 | 75.0 | 43.1 |
| Income €500–1,000 | 67.5 | 72.2 | 35.2 |
| Income €1,000–3,000 | 88.8 | 59.2 | 25.5 |
| Income > €3,000 | 93.7 | 44.3 | 18.2 |

|  |  |  |  |
| --- | --- | --- | --- |

Table A3. Model Fit Indices for Logistic Regressions

| Outcome | | Nagelkerke’s R² | Hosmer-Lemeshow *p*-value | AUC |
| --- | --- | --- | --- | --- |
| Anxiety Disorder | 0.187 | 0.504 | 0.719 |  |
| Affective Disorder | 0.177 | 0.442 | 0.716 |  |
| Trauma-related Disorder | 0.196 | 0.544 | 0.720 |  |
| Substance Use Disorder | 0.216 | 0.931 | 0.750 |  |

Table A4. Environmental and Disclosure Risk Factors for Mental Disorders

| Factor | | Associated Effect | Outcome | OR 95% CI | *p*-value |
| --- | --- | --- | --- | --- | --- |
| Work in Client’s Apartment | Increased Risk | Trauma-related Disorder | 2.23 [1.38, 3.61] | 0.001 |  |
| Work in Client’s Apartment | Increased Risk | Affective Disorder | 2.41 [1.45, 4.03] | 0.001 |  |
| Work in Client’s Apartment | Increased Risk | Anxiety Disorder | 1.64 [1.02, 2.63] | 0.040 |  |
| Work in Client’s Apartment | Increaed Risk | Substance Use Disorder | 2.37 [1.36, 4.11] | 0.002 |  |
| Escort Work | Reduced Risk | Affective Disorder | 0.51 [0.30, 0.88] | 0.015 |  |
| Disclosure to Partner | Reduced Risk | Anxiety Disorder | 0.53 [0.33, 0.85] | 0.009 |  |
| Disclosure to Social Contacts | Increased Risk | Anxiety Disorder | 2.08 [1.28, 3.39] | 0.003 |  |

**Appendix B.**

The following items were extracted from the self-developed Sex-Work Questionnaire used in this study. Only sociodemographic and occupational questions relevant to the present analyses are presented here.

**Appendix B1 Short Version of the Sex-Work Questionnaire (Sociodemographic and Occupational Items)**

|  | What is your gender? | Female  Male  Divers |
| --- | --- | --- |
|  | Year of birth? |  |
|  | What nationality/ nationalities do you have? |  |
|  | Migration background? | Yes  No |
|  | Do you speak German? | Yes  No |
|  | Are you homeless? | Yes  No |
|  | Do you have a school degree? | Yes  No |
|  | Have you completed vocational training or a degree? | Yes  No |
|  | Are you currently working in sex work? | Yes  No |
|  | Are you in a (steady) relationship? | Yes  No |
|  | *If yes, does your partner know about your activity in the context of sex work? | Yes  No |
|  | Approximately what is your monthly income? | I have no income of my own  under 500 €  under 1000 €  €2000 to €3000  3000 € or more |
|  | Where do you usually meet/ look after your customers/ clients?  *(Multiple answers are possible)* | Online  Hotel  Brothel  Own Apartment  Street-based  Escort Services  Client’s Apartment |
|  | Do you operate under your real name? | Yes  No |
|  | Do you have social contacts outside the context of sex work? | Yes  No |
|  | *If yes, do they know about your work? | Yes  No |
|  | Have you been forced into sex work? | Yes  No |

*(Note: A complete English version of all questionnaires is available from the corresponding autor upon request.)*
